# Long Island Veterinary Society

**Published:** 1890-07

**Authors:** 


*Long Island Veterinary Society.*—A regular meeting of the Long Island Veterinary Society was held on June 18th, 1890, at No. 74 Adams Street, the President, Dr. George H. Berns, in the chair.

The following members were present: Drs. Berns, Bowers, Newman, Buckley, Breslin, Housman, Pendry.

The minutes of the previous meeting were read and approved. Board of Censors made no report. Committee on State Legislation reported progress. The next order of business being reading of papers, Dr. Thomas M. Buckley read an interesting paper, entitled "Veterinary Hygeiology and Antiseptics." He said: "Being accorded the privilege and pleasure of reading before the Society this evening a paper on 'Veterinary Hygeiology and Antiseptics,' I beg to say the last can be but an apology, as it is by far too great and important a subject to attempt to do anything like justice to on such an occasion as this. Increasing observation satisfies me that there remains a vast amount for us, as veterinarians, to accomplish in order to carry out hygienic conditions. Good quarantine laws should be enforced wherever and whenever an outbreak is discovered, and the present Bureau of Animal Industry and its efficient officers deserve all praise for the noble work they are quietly but surely accomplishing. The illimitable demand for animal food by rich and poor is, perhaps, in no part of the world greater than in the United States, and when we take into consideration the variety of diseases which are transmitted to man from the animal kingdom—one especially, *tuberculosis*—and not only may it be transmitted to man, but to any species of animal that partakes of raw flesh in which is contained tuberculous matter; and when we take into consideration the vast amount of milk that forms the principal daily article of diet for the infant and invalid, whose condition and low resisting powers render them especially susceptible to absorption of poisons, it is no wonder that our mortality by tuberculosis is so great, as statistics show for the city of Brooklyn: From January 1st to December 31st, 1880, 1736; from January 1st to December 31st, 1881, 1754; from January 1st to December 31st, 1882, 1806; from January 1st to December 31st, 1883, 1847; from January 1st to December 31st, 1884, 1913; from

January 1st to December 31st, 1885, 1995; from January 1st to December 31st, 1886, 2085; from January 1st to December 31st, 1887, 2026; from January 1st to December 31st, 1888, 2051; from January 1st to December 31st, 1889, 2055; from January 1st to June 7th, 1890, 1018. Total, 20,286. I firmly believe that this great mortality can be influenced by the co-operation of the veterinary profession in reporting any suspicious case to the inspectors, thereby aiding the bureau, whose staff is entirely inadequate for the amount of territory to be covered. Under the head of Stable Hygiene he concisely enumerated a few essential points: Stables should be spacious, well ventilated, thoroughly drained and free from dampness. No bedding or manure should be permitted to remain in the stall in the daytime, and, unless absolutely unavoidable, should a sick animal be treated in cellar stables. Stables should be limewashed at least four times a year, and the flooring should be washed down weekly during the Summer months with one of the standard disinfecting solutions. The majority of the ultimate results of all surgical cases would be far more successful if we adopted rigid antiseptic surgery. Carbolic acid, biniodide of mercury, bichloride of mercury, iodoform, boracic acid, chloral hydrate, peroxide of hydrogen and creolin are the most important agents, and by strict adherence of the surgeon to aseptic principles his painstaking and labor will not be in vain."

